# Brain damage and behavioural disorders in fish induced by plastic nanoparticles delivered through the food chain

**DOI:** 10.1038/s41598-017-10813-0

**Published:** 2017-09-13

**Authors:** Karin Mattsson, Elyse V. Johnson, Anders Malmendal, Sara Linse, Lars-Anders Hansson, Tommy Cedervall

**Affiliations:** 1 Department of Biochemistry and Structural Biology, Lund University, P.O. Box 124, SE-221 00 Lund, Sweden; 2 NanoLund, Lund University, SE-221 00 Lund, Sweden; 3 CytoViva, Inc. 570 Devall Drive, Auburn, AL 36832 USA; 4 Department of Biology/Aquatic Ecology, Lund University, SE-223 62 Lund, Sweden

## Abstract

The tremendous increases in production of plastic materials has led to an accumulation of plastic pollution worldwide. Many studies have addressed the physical effects of large-sized plastics on organisms, whereas few have focused on plastic nanoparticles, despite their distinct chemical, physical and mechanical properties. Hence our understanding of their effects on ecosystem function, behaviour and metabolism of organisms remains elusive. Here we demonstrate that plastic nanoparticles reduce survival of aquatic zooplankton and penetrate the blood-to-brain barrier in fish and cause behavioural disorders. Hence, for the first time, we uncover direct interactions between plastic nanoparticles and brain tissue, which is the likely mechanism behind the observed behavioural disorders in the top consumer. In a broader perspective, our findings demonstrate that plastic nanoparticles are transferred up through a food chain, enter the brain of the top consumer and affect its behaviour, thereby severely disrupting the function of natural ecosystems.

## Introduction

The production of plastic material has increased tremendously during the last decades^1, 2^, and about 10% of the plastics produced annually end up in the oceans^3^ through sewage treatment plants, waste handling or aerial deposition^4^, constituting 60–80% of the total marine debris^5^. Plastic debris has been shown to affect over 660 marine species^6^ through entanglement and ingestion, and is thus a severe and potent pollutant in aquatic environments^6^. Once in the aquatic environment, plastic material breaks up into smaller pieces through the action of sunlight, waves, living organisms in the water and by the water itself ^7, 8^. Eventually plastic material is broken down to nanoparticles^9–12^, which may be an even more potent threat since plastic nanoparticles are able to pass through biological barriers^13^, penetrate tissues^14^ and accumulate in organs^15^ and affect behaviour and metabolism of organisms^16, 17^. Those effects are generally not due to the toxicity of the material *per se*, but rather a result of the physical features of the nanoparticles. Hence, the particle size plays a pivotal role for their biological impact^18^ as size affects the curvature and provide a large surface area^18, 19^. Smaller particles are generally more toxic than the corresponding bulk material at the same mass concentration^20–22^, and the mobility, biological fate and bioavailability depend on size, shape, charge and other nanoparticle properties^23, 24^.

The freshwater invertebrate *Daphnia magna* can ingest nano- and micro-sized (20 nm to 70 μm) particles from water^21, 25–27^, are commonly used in toxicity studies^28^ and has a pivotal role in many food chains^16, 17, 29^. Previous work has shown that the uptake rate depends on particles size^25, 30, 31^ and charge^32^. For example, *Daphnia magna* were shown to have a lower uptake rate of 20 nm than 1000 nm polystyrene particles, although when compared at equivalent surface area, the uptake was equal or higher for the small particles. Furthermore, indirect intake rate via algal food was higher than direct intake from water^30^. The precise manner in which particle size, charge and surface area affect the intake and biological impact of nanoparticles is, however, still unknown.

Here we report novel findings on how plastic nanoparticles strongly affect an aquatic food chain from the zooplankter *Daphnia magna* to the top consumer, the freshwater fish, Crucian carp (*Carassius carassius*), which is common in anthropogenically affected waters. We exposed *Daphnia magna* to a range of polymeric nanoparticles directly in water or via algae (*Scendesmus sp*.). We show that positively charged amino modified polystyrene nanoparticles affect both *Daphnia* and top consumer (fish) in a size dependent manner. We also show that the nanoparticles were transferred through a three-level food chain from algae through zooplankton to fish, which showed behavioural disorders. Moreover, those behavioural disorders depended on the size of the nanoparticles and analyses by hyperspectral microscopy showed that the plastic nanoparticles were present in the fish brains. Hence, we here, for the first time, demonstrate the mechanistic chain from uptake of nanoplastic particles by algae, through transport up the food chain and, finally, effects on the brain physiology and behaviour of top consumer (fish). On a broader scale such effects are likely to considerably affect natural ecosystems, since top predators have a crucial impact on lower trophic levels and ecosystem functions^33^.

## Results

### Effects on Daphnia magna

**Figure 1:**
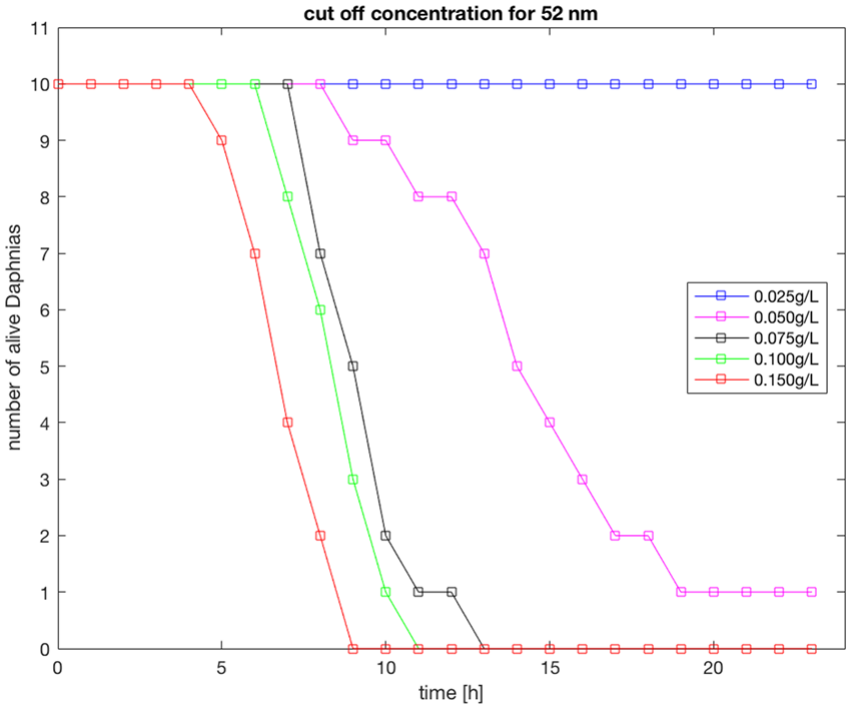
Mortality after exposure. Number of alive *Daphnia magna* 0–24 h after exposure to different concentrations (0.025–0.150 g/L) of 52 nm amino modified polystyrene nanoparticles. The figure shows that *Daphnia* mortality rates depend on the concentration of the particles, n = 10 for each concentration.

Out of the tested nanoparticles of different size and charge (Table 1) only amino-modified positively charged polystyrene nanoparticles with a diameter of 52 nm affected *Daphnia*, whereas larger particles of the same material (120–330 nm) had no effect on the animals and we therefore focused on this particle size in our study. For the 52 nm particles, the toxicity was strongly dependent on the particle concentration. Up to a concentration of 0.025 g/L all *Daphnia* were still alive after 24 hours, and above 0.075 g/L all were dead within 13 h (Fig. 1). Comparing the toxicity of differently sized amino-modified polystyrene nanoparticles, where either the mass, surface area or the number of particles were the same, revealed that size was the only important factor for toxicity. To rule out a potential batch dependent toxicity of 52 nm amino modified polystyrene particles, the same type of particles but in a size of 53 nm, 57 nm as well as 58 nm were also tested.

**Table 1:** Characteristics for particles tested for toxicity to *Daphnia magna*, including particle name, diameter and concentration.

Particle	Diameter size (nm)	Concentration (g/L)	Surface charge
PS-NH_2_ Amino-modified	52, 53, 57, 58, 120, 180, 330	0.005, 0.010, 0.025, 0.050, 0.075, 0.10, 0.15	positive
PS-COOH Carboxylate	26^1^, 60^1^, 92, 160, 190, 220	0.025, 0.050, 0.075, 0.10, 0.2, 0.4	negative
PS-OSO_3_H Sulfonated	25, 200	0.025, 0.050, 0.075, 0.10, 0.2, 0.4	negative
PMMA	68, 140	0.025, 0.050, 0.075, 0.10, 0.2, 0.4	negative

Only the PS-NH_2_ amino modified particles was found to be toxic to *Daphnia* and we therefore performed our fish study using this type of particles. ^1^Two different batches were tested.

**Figure 2:**
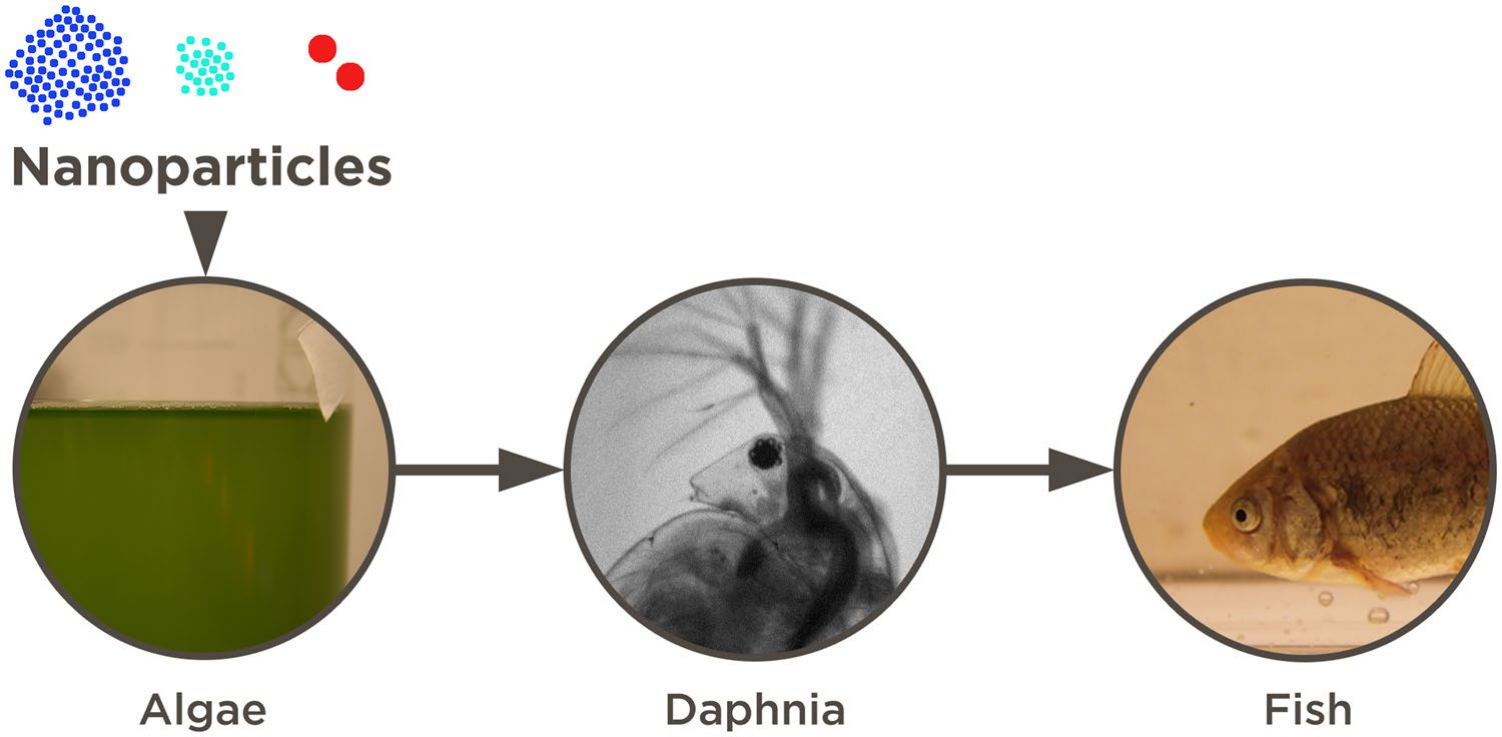
Food chain. Food chain from algae-zooplankton-fish, nanoparticles (53 nm mass (dark blue), 53 nm surface area (light blue) and 180 nm (red)).

**Figure 3:**
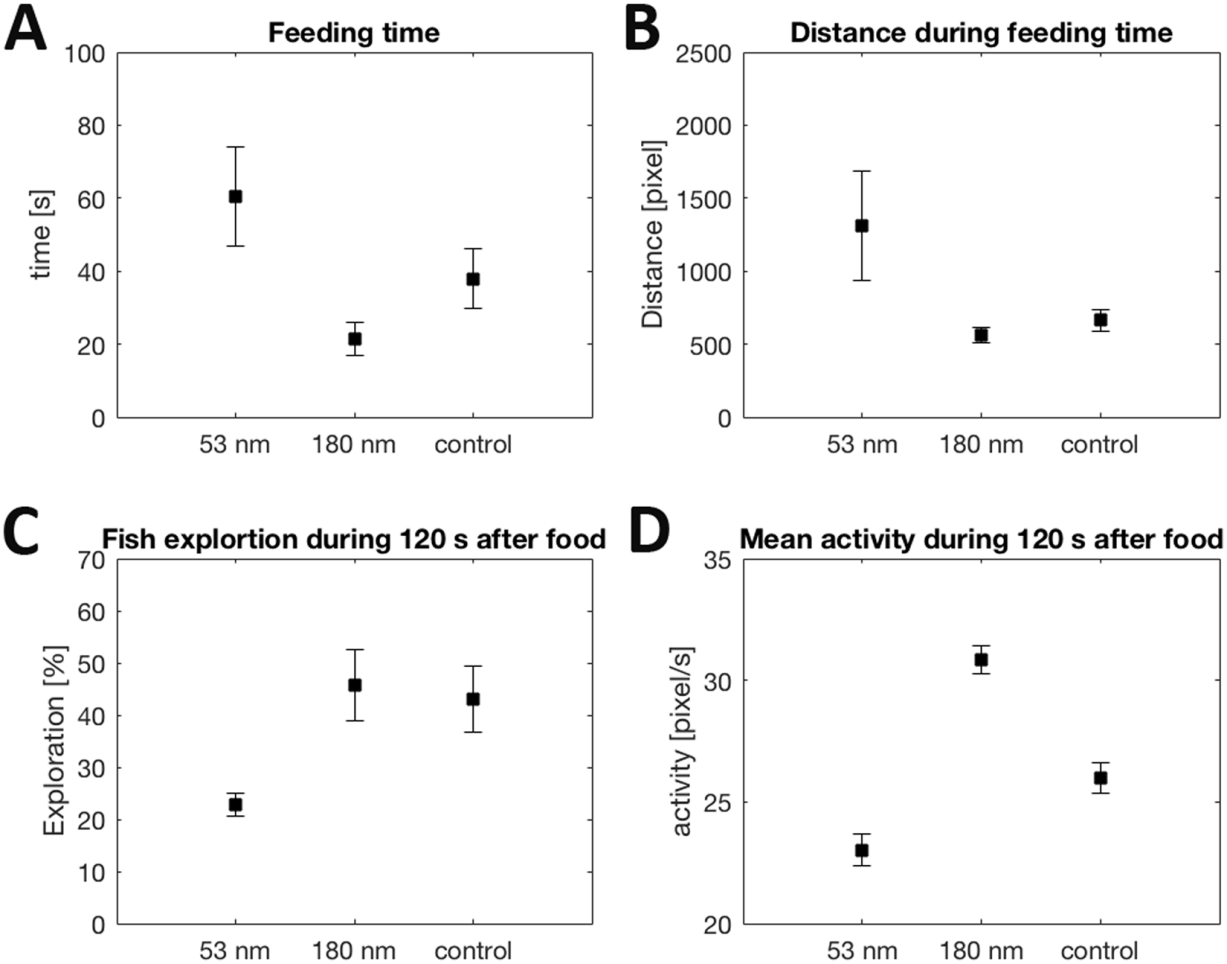
Top consumer response. Fish exposed to amino modified polystyrene nanoparticles of different sizes delivered through a food chain of algae and zooplankton behave differently. (**A**) fish feeding time (s) after exposure to different sizes of nanoparticles, p < 0.030 with ANOVA post hoc between 53 nm and 180 nm, (**B**) swimming distance during feeding time, p < 0.018 with ANOVA post hoc between 53 nm and 180 nm, (**C**) fish exploration within each aquarium during the first 120 s of the feeding time (**D**) mean activity (px/s) during the first 120 s of the feeding time, p < 0.001 with ANOVA post hoc between all groups. Data are shown for control fish that were not exposed to nanoparticles (n = 6 aquaria), 180 nm nanoparticles (n = 6 aquaria), and 53 nm particles with the same mass concentration as 180 nm particles (n = 5 aquaria). All graphs show the mean ± SE. The graphs shown that fish fed with 53 nm nanoparticles had a longer feeding time, that they swam a longer distance to feed and that they explored less area of the aquaria, as well as that they had a lower activity compared to fish receiving 180 nm particles and control fish. Moreover, fish fed with 180 nm particles were the fastest feeders and had the highest activity.

Equipped with the insight obtained from *Daphnia magna*, we next set out to study the effects of plastic nanoparticles (53 nm and 180 nm) on the entire food chain and if the effects of nanoparticles are transferred to the fish in a food chain starting from algae (Fig. 2). Analyses of fish feeding times – the time it took for each group (aquarium) to consume 50% of the provided *Daphnia* – showed that the fish receiving 53 nm particles ate more slowly than the control fish. Contrary to our expectations, fish fed with 180 nm particles were the fastest feeders (Fig. 3A; p < 0.030 ANOVA). Furthermore, detailed analyses of the hunting behaviour showed that the fish fed with 53 nm particles swam a longer distance to eat 50% of the provided zooplankton (Fig. 3B, p < 0.02 ANOVA), and explored less space within each aquarium (Figs 3C and S1). They also had a significantly lower activity (px/s), in contrast to the fish fed 180 nm particles which instead showed a higher activity than controls (Fig. 3D; p < 0.001 ANOVA). In natural systems, a slower feeding rate combined with a longer swimming distance before successful feeding likely leads to suboptimal energy use and a more pronounced exposure to predation. Collectively, these consequences point to considerable effects on fitness and thereby on ecosystem function for fish exposed to plastic nanoparticles of about 50 nm size.

**Figure 4:**
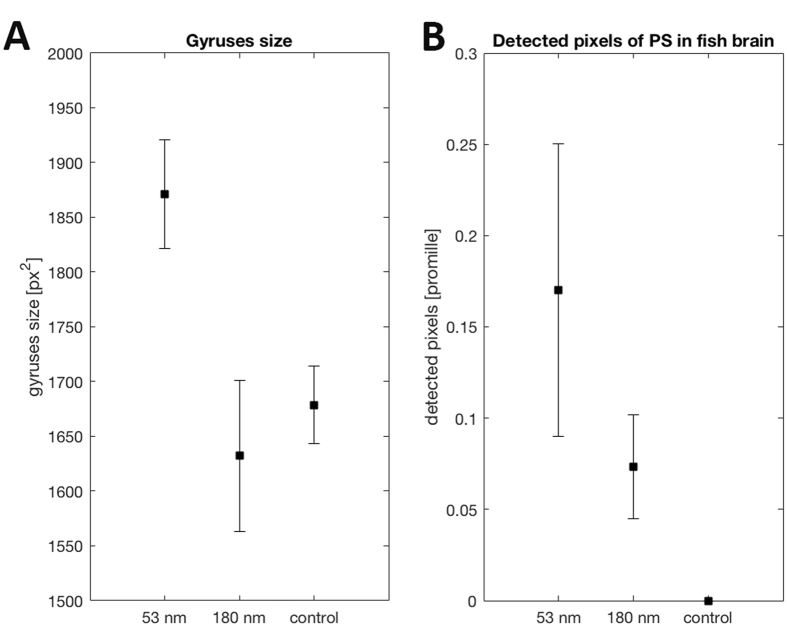
Brain effects. (**A**) Measured gyri size (px^2^) of fish brains after exposure to different sizes of polystyrene nanoparticles, including 53 nm (n = 7), 180 nm (n = 11) and control (n = 15) which was not exposed to any nanoparticles. (**B**) number of detected pixels corresponding to polystyrene particles in homogenized fish brain samples, (n = 3 for each treatment). Fish receiving 53 nm nanoparticles had significantly larger gyri size than the 180 nm group, p < 0.021 ANOVA with Turkey’s post hoc. Polystyrene was detected in all brains from fish exposed to nanoparticles, whereas no polystyrene was detected in the control group.

The observed behavioural changes in the fish suggest that their brains were affected by the particles. To confirm this, we explored the unique spectral features of tracking polystyrene using a CytoViva Hyperspectral Imaging System to obtain a wavelength spectrum from each pixel of the light scattered from fish brains. Polystyrene was detected in the brains from all analysed fish fed with polystyrene nanoparticles, whereas no polystyrene was detected in brains from control fish (Figs 4B and S2).

We also found that fish exposed to nanoparticles had a higher weight loss and less water in their brains than control fish (Fig. S3). Moreover, the microscope imaging shows that the gyri in the cerebral lobes were larger in fish exposed to 53 nm particles (Fig. 4A; p < 0.025 ANOVA), suggesting also morphological effects from those particles. Hence, the morphological changes in the brains of the 53-nm-sized nanoparticle-fed fish suggest that fish brains were directly affected by the plastic nanoparticles and that the effects depended on particle size. Taken together, our results suggest that some of the plastic nanoparticles fed to fish through a food web end up in the fish brains. Furthermore, we unravel a mechanistic link between behavioural disorders and the incorporation of nanoparticles in brain tissue.

## Discussion

The amount of plastics in the world’s water bodies is rapidly increasing and this material degrades in size over time and will eventually break down into plastic nanoparticles. Due to their small size, they easily enter the basis of natural food chains, although it is unclear how these particles affect aquatic ecosystems. We show here that 52 nm positively charged amino modified polystyrene nanoparticles are toxic to *Daphnia* and that fish feeding on *Daphnia* containing plastic nanoparticles change their behaviour in terms of activity, feeding time and the distance they need to swim to consume their provided food. Furthermore, the behavioural changes depend on the size of the particles. However, fish receiving 180 nm particles were differently affected as they were the fastest feeders and had the highest activity. In nature, the particles likely become aggregated with biological or inorganic material, but we here show that the nano-size effect remains after passing through the *Daphnia* digestive system. For example, Ward *et al*.^34^ exposed the blue mussel *Mytilus edulis* and the oyster *Crassostrea virginica* to polystyrene nanoparticles, aggregated nanoparticles and micro-particles and found a higher ingestion rate for the aggregated nanoparticles^34^. Wegner *et al*.^35^ exposed the mussel *Mytilus edulis* to polystyrene nanoparticles both as nano-sized particles and as aggregated polystyrene nanoparticles. They found a reduced filtering rate and an increased production of pseudofeces^35^. In this context, our results point to an acute need for a deeper understanding of the size-dependent toxicity effects of nanoparticles when released into nature. How these particles affect organisms higher up in the food web, such as fish, as well as how they affect birds and mammals are unclear. In 2015, the estimated amount of plastics being released into the ocean was between 4.8 and 12.7 million tons, with a steady increase the coming years^36^. Eventually this plastic will degrade in size and reach the nanometer size range.

Here we demonstrate how plastic nanoparticles are transported up the food chain and are detected in brain tissue of the fish top consumer whereas no polystyrene were detected in the control group. Moreover, we also here report macroscopic changes in the brain structure and water content in fish that have received plastic nanoparticles. By using hyperspectral microscopy, we were able to detect polystyrene particles in fish brain tissue and thereby we have, for the first time, demonstrated that the plastics nanoparticles can be transported across the blood-brain barrier in fish. Moreover, this result suggests a mechanistic link between the observed behavioural changes and the presence of plastic nanoparticles in the brain tissue. In the present study, we observed changes in the brain which may have been caused by specific interactions between the plastics and the brain tissue, although we cannot rule out that other organs may also be affected. Our study lasted for two months, but during the first half of the experiment we observed no changes in behaviour of the nanoparticle fed fish, suggesting that fish are affected by the particles that are accumulated in the fish. In nature, the *Daphnia* and fish are likely exposed to low concentrations of plastic nanoparticles during their whole life-time, which allows accumulation processes to act for a much longer time period than in our study, since fish, such as crucian carp, may live for more than 10 years^37^. However, our results also imply that effects on biota from plastic nanoplastics are dependent on both concentration and size of the particles, which opens up for manufacturers to adjust production of nanoparticles to sizes that are less hazardous to organism metabolism and thereby ecosystem function.

The main conclusion from our study is that plastic nanoparticles are transferred through three tropic levels, suggesting that they are likely to be transferred even further up the food web to ultimately reach humans, the top-level consumer. Hence, in a broader perspective, our results may have implications for human wellbeing, although such consequences of the accelerating disposal rate of plastics is yet not well recognized or understood.

## Materials and Methods

### Testing for suitable particles with *Daphnia magna*

Polystyrene particles with different surface modifications, charges, sizes (25 nm to 330 nm) and at a range of concentrations (0.005 g/L to 0.150 g/L) were tested for toxicity towards *Daphnia magna*. On day 1, 100 ml algae or water were added to each bottle together with particles with different concentrations except for the control bottle, which received only water (Table 1). The bottles were then shaken for 2 minutes, and the algae were allowed to ingest the particles for 24 hours. On day 2, 900 ml water was added to the algae/water together with 10 adult *Daphnia magna* with an approximate size of 3 mm. The number of dead *Daphnia* was counted every hour for 24 hours and the bottles were then gently stirred to distribute the algae or water evenly. To rule out a potential batch dependent toxicity of 52 nm amino modified polystyrene particles, the same type of particles but in a size of 53 nm, 57 nm as well as 58 nm were also tested.

### Nanoparticle preparation and characterization

Positively charged amino-modified polystyrene (PAO_2_N) particles with diameters of 52 nm, 53 nm, 57 nm, 58 nm, 120 nm, 180 nm and 330 nm were purchased from Bang laboratories (Fisher, IN, USA). The particles were dialyzed with fresh tap water for 24 hours. The particle size was measured, to ensure that particles size remained constant during the experiment, with Dynamic Light Scattering (DLS) both before and after dialysis, as well as one week after the dialysis. No change in particle size was recorded during the study. We chose to not use surface labelled particles since this may affect the surface chemistry. Moreover, it is unknown how passage through the digestive systems of *Daphnia* and especially fish might affect the labelling and vice versa.

### Fish experiment

Two sizes of particles were chosen for the fish experiment, one with the size of 53 nm that was shown to affect the *Daphnia* and one larger, 180 nm, that did not show any toxicity towards *Daphnia*. The particle size were confirmed with DLS and measured 56 nm (PI: 27%) and 174 nm (PI: 18%) in the water used during the experiment. Twenty-four aquaria with three fish in each were divided into four groups. The first group (the 180 nm group) received 180 nm particles at a concentration of 0.1 g/L. The second group received the same mass concentration (0.1 g/L) of 53 nm particles (the 53 nm mass group). The third group also received 53 nm particles, but at a lower concentration corresponding to the same surface area as the group receiving 180 nm particles (the 53 nm surface area group, concentration 0.029 g/L). The results from this treatment are, for clarity, presented in Supplementary material, (Table S1). The fourth group, the control group, did not receive any nanoparticles. All fish were measured and weight before the experiment started. The study was performed under the permission from the Malmö/Lund Ethical committee (D nr 14 13–12) and was performed according to the current laws in Sweden.

### Food chain

Algae (*Scenedesmus sp*.) with a diameter of approximately 25 μm were cultivated in aquaria. On day 1, 500 mL algae with a concentration of 450 $\mu g/L$ were mixed with water and particles to a total volume of 1 L in four different test bottles (except for the control bottle, which received only water). After 24 hours *Daphnia magna* (20 *Daphnia*/fish) were added to the algae medium. After 2 hours, the *Daphnia* were collected on a net with a mesh size of 150 $\mu m$ and washed two times with 150 mL water. Each fish (Crucian carp, *Carassius carassius*) was then served 20 *Daphnia*, i.e. 60 *Daphnia* per aquarium.

We replicated this natural food chain such that the fish eventually ingested, via algae and *Daphnia*, the same type of amino-modified polystyrene nanoparticles as used for the *Daphnia* toxicity with diameters of 53 nm and 180 nm (Fig. 2). To distinguish between size and mass effects, two concentrations of the 53 nm particles were used, one that corresponded to the same surface area and one that corresponded to the same mass as the 180 nm particles. The three groups: 180 nm, 53 nm surface area (Table S1) and 53 nm mass were studied together with the control group, that did not receive any nanoparticles. Sixty *Daphnia* individuals were introduced as food to each fish aquarium every third day for a period of 67 days.

### Video analysis

On day 62, we monitored the hunting behaviour of the fish by video recording each aquarium separately during 2 minutes before the fish received food and 10 minutes after. Since the smaller particles were toxic to the *Daphnia* and thereby possibly affected their interaction with the fish, all groups of fish were on the 62^nd^ day fed with *Daphnia* that had not received any nanoparticles. Each fish position was registered each second during the whole tracking period using the software ImageJ. The feeding time – the time it took for the fish to consume 50% of the provided food *(Daphnia)* – was registered. An ANOVA post hoc was used to test differences between treatments.

### Brain analysis

On day 64, all fish were collected and anaesthetized using benzocaine. They were measured and weighed before the neck was cut and the brain was extracted. All samples were stored at −80 °C. The brain was weighed and an image was recorded with Olympus SZX7 microscope with an Infinity 1 camera and then freeze-dried and weighed again before it was homogenized in PBS buffer. The area of two gyri in all brain images was measured in pixels^2^ using ImageJ and further calculated with Matlab. Finally, three brains from each group were analysed with CytoViva hyperspectral microscope. This microscope was equipped with an enhanced darkfield illuminator and visible-near infrared (400–1000 nm) hyperspectral imaging components. The homogenized brain samples were imaged under 60x magnification. Each image captured one pixel line at a time using an automated stage. These pixel lines were compiled to form hyperspectral images, also known as datacubes, which contain spatial and spectral data for each pixel. For each image of exposed brain that was acquired, a spectral library corresponding to polystyrene was created. This was accomplished by gathering several regions of interest from each exposed brain image and filtering the spectra associated with those regions against 3 negative control images (homogenized brain with no polystyrene). Any spectra that matched spectra in the negative controls were eliminated from the polystyrene spectral libraries. Then, the polystyrene was spectrally mapped and identified in the exposed brain images using the Spectral Angle Mapper algorithm.

## Electronic supplementary material

Supplementary information
