# Supplementary material for: Brain damage and behavioural disorders in fish induced by plastic nanoparticles delivered through the food chain
Source: Sci Rep. 2017 Sep 13;7:11452. doi: 10.1038/s41598-017-10813-0 (PMC5597631; doi:10.1038/s41598-017-10813-0)
Supplement: Supplementary file 1 — Supplementary information [file 41598_2017_10813_MOESM1_ESM.pdf]

SUPPLEMENTARY INFORMATION

Brain damage and behavioural disorders in fish induced by plastic nanoparticles delivered through the food chain

Authors

Karin Mattsson[1,2], Elyse V. Johnson[3], Anders Malmendal[1], Sara Linse[1,2], Lars-Anders Hansson[2,4], Tommy Cedervall[1,2]

Table S1. Effects from 53 nm polystyrene particle concentrations based on surface area. In order to distinguish between size and mass effects, two concentrations of the 53 nm particles were used, one that corresponded to the same surface area and one that corresponded to the same mass as the 180 nm particles. The effects of 53 nm particles calculated based on the same mass as 180 nm are given in fig. 2 and 3. Below we show the effects of 53 nm particles when delivered based on similar surface area as 180 nm particles. The table shows effects on feeding time, distance during feeding, exploration, activity and brain gyruses size of the fish top consumer (*Carassius carassius*).

	53 nm mean (±SD)
Feeding time (s)	57.8 (0.842)
Distance during feeding (pixel)	1860 (420)
Exploration (%)	47.6 (9.15)
Activity (pixel/s)	31.0 (0.842)
Gyruses size (pixel^2)	1650 (69.4)
Detected pixels (‰)	0.16 (0.080)

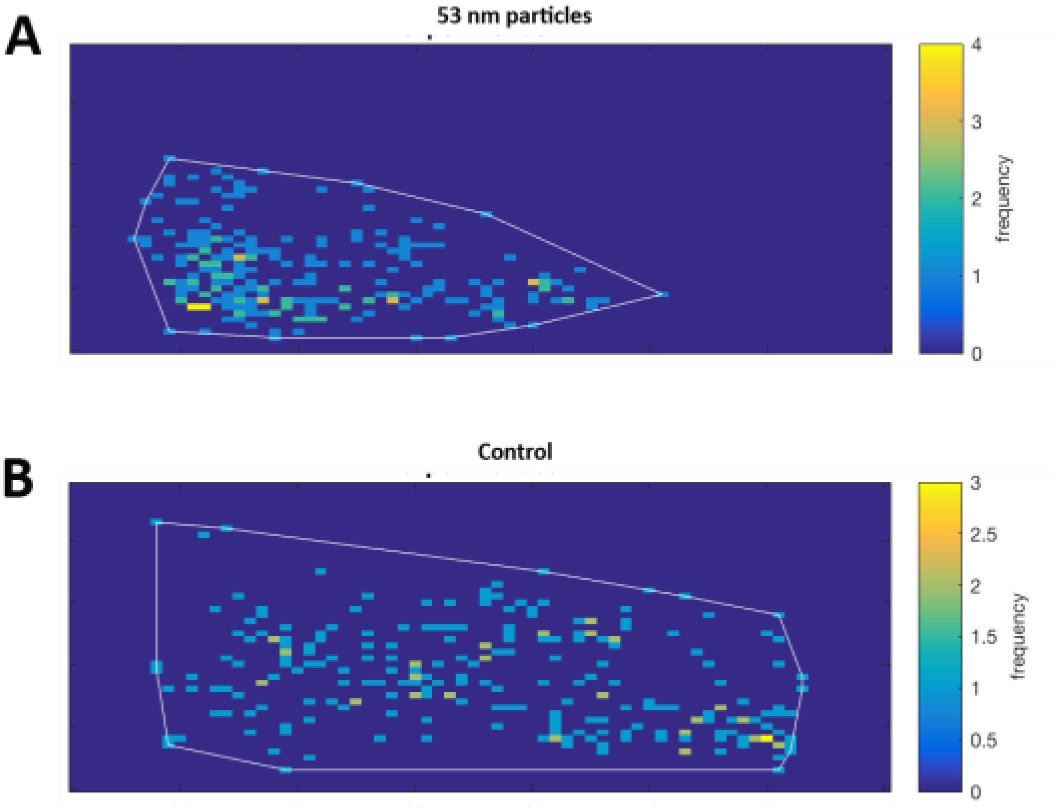

Figure S1: Exposed fish explore less. Detailed fish exploration of the aquaria during the first 120 s of the feeding time (data for all aquaria shown in figure 2c). a) Fish exploration from 53 nm mass group aquaria from median value aquaria in figure 2c and b) Fish exploration from control group, aquaria median value in figure 2c.

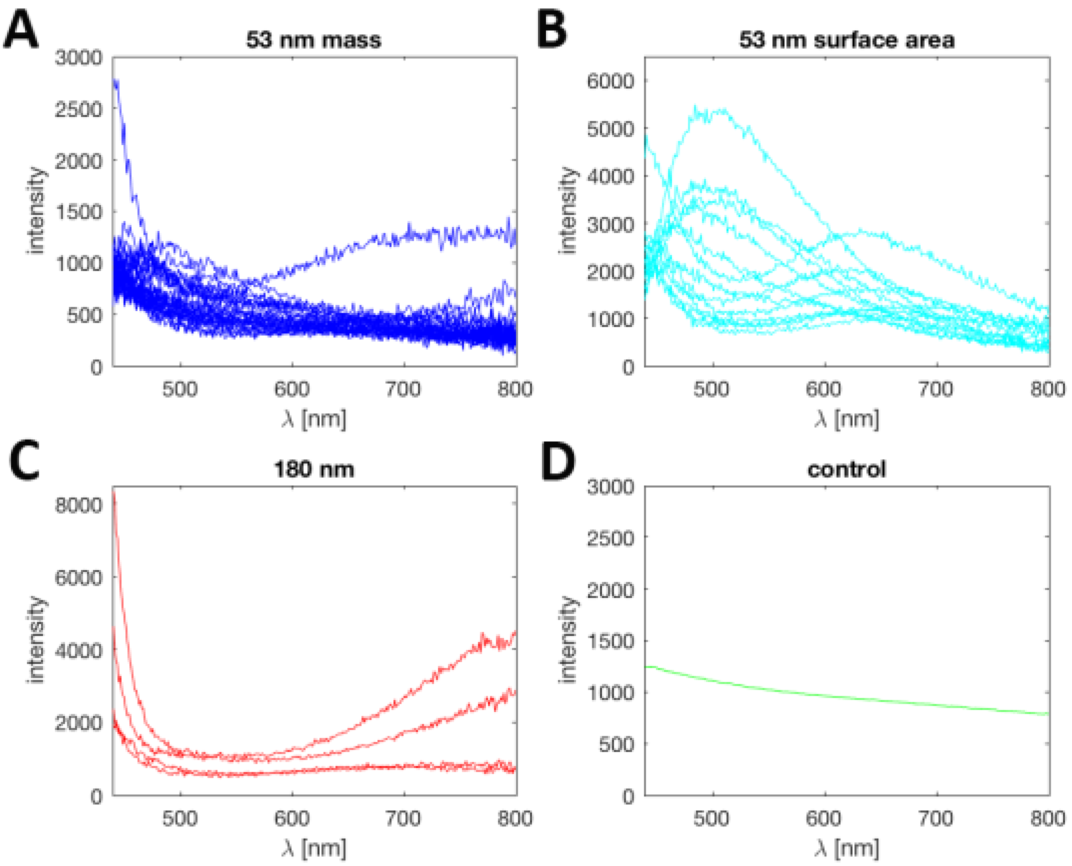

Figure S2: Nanoparticles detected in top consumer brains. Spectral analysis reveals polystyrene in brains of fish fed with nanoparticles. Fish brain spectra for a) 53 nm mass b) 53 nm surface area, c) 180 nm, and d) control group. n=3 in all treatments.

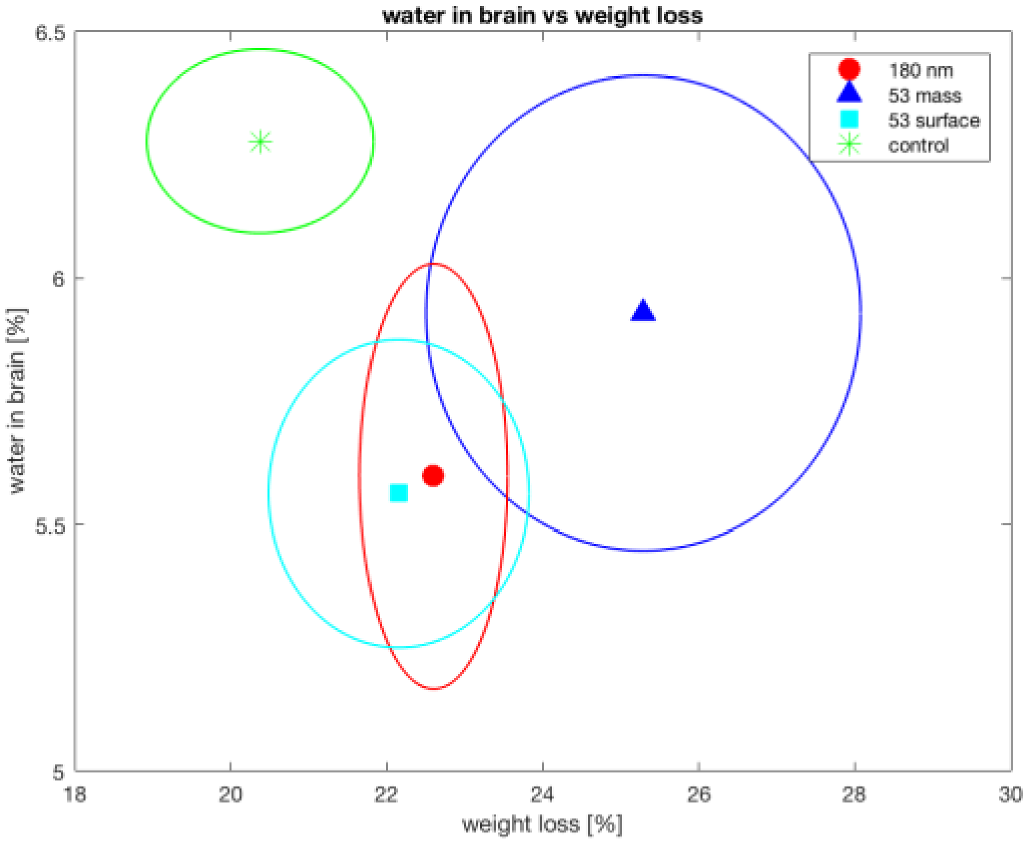

Figure S3: Brain morphology changes. Water in brain vs weight loss for the four groups; standard errors of the means as circles around the dots. Exposure to nanoparticles led to weight loss and morphological changes in fish brains.
